# Analysis of drug resistance among difficult-to-treat tuberculosis patients in Ghana identifies several pre-XDR TB cases

**DOI:** 10.3389/fmicb.2022.1069292

**Published:** 2023-01-12

**Authors:** Isaac Darko Otchere, Portia Abena Morgan, Prince Asare, Stephen Osei-Wusu, Samuel Yaw Aboagye, Stephen Ofori Yirenkyi, Abdul Basit Musah, Emelia Konadu Danso, Georgina Tetteh-Ocloo, Theophilus Afum, Adwoa Asante-Poku, Clement Laryea, Yaw Adusi Poku, Frank Bonsu, Sebastien Gagneux, Dorothy Yeboah-Manu

**Affiliations:** ^1^Noguchi Memorial Institute for Medical Research, College of Health Sciences, University of Ghana, Accra, Ghana; ^2^Institute for Environment and Sanitation Studies, College of Basic and Applied Sciences, University of Ghana, Accra, Ghana; ^3^Eastern Regional Hospital, Koforidua, Ghana; ^4^Chest Department, 37 Military Hospital, Accra, Ghana; ^5^National Tuberculosis Control Program, Ghana Health Service, Accra, Ghana; ^6^Medical Parasitology and Infection Biology, Swiss Tropical and Public Health Institute, University of Basel, Basel, Switzerland

**Keywords:** pre-XDR-TB, drug resistance, Ghana, monitoring, tuberculosis, screening, treatment

## Abstract

**Background:**

Resistance to tuberculosis (TB) drugs has become a major threat to global control efforts. Early case detection and drug susceptibility profiling of the infecting bacteria are essential for appropriate case management. The objective of this study was to determine the drug susceptibility profiles of difficult-to-treat (DTT) TB patients in Ghana.

**Methods:**

Sputum samples obtained from DTT-TB cases from health facilities across Ghana were processed for rapid diagnosis and detection of drug resistance using the Genotype MTBDR*plus* and Genotype MTBDR*sl*.*v2* from Hain Life science.

**Results:**

A total of 298 (90%) out of 331 sputum samples processed gave interpretable bands out of which 175 (58.7%) were resistant to at least one drug (ANY^r^); 16.8% (50/298) were isoniazid-mono-resistant (INH^r^), 16.8% (50/298) were rifampicin-mono-resistant (RIF^r^), and 25.2% (75/298) were MDR. 24 (13.7%) of the ANY^r^ were additionally resistant to at least one second line drug: 7.4% (2 RIF^r^, 1 INH^r^, and 10 MDR samples) resistant to only FQs and 2.3% (2 RIF^r^, 1 INH^r^, and 1 MDR samples) resistant to AMG drugs kanamycin (KAN), amikacin (AMK), capreomycin (CAP), and viomycin (VIO). Additionally, there were 4.0% (5 RIF^r^ and 2 MDR samples) resistant to both FQs and AMGs. 81 (65.6%) out of 125 INH-resistant samples including INH^r^ and MDR had *katG*-mutations (MT) whereas 15 (12%) had *inhApro*-MT. The remaining 28 (22.4%) had both *katG* and *inhA* MT. All the 19 FQ-resistant samples were *gyrA* mutants whereas the 10 AMGs were *rrs* (3), *eis* (3) as well as *rrs*, and *eis* co-mutants (4). Except for the seven pre-XDR samples, no sample had *eis* MT.

**Conclusion:**

The detection of several pre-XDR TB cases in Ghana calls for intensified drug resistance surveillance and monitoring of TB patients to, respectively, ensure early diagnosis and treatment compliance.

## Introduction

Tuberculosis (TB) continues to be a global health threat and a leading cause of adult mortality by a single infectious disease over the years and is currently is second only to COVID-19 (WHO, 2020a; Woolf et al., 2021). The WHO recommends the directly observed treatment short-course (DOTS) regimen, which involves the use of multiple antibiotics in an intensive phase therapy (Isoniazid; INH, Rifampicin; RIF, pyrazinamide; PZA, and ethambutol; EMB) for 2 months followed by 4 months continuous-phase administration of INH and RIF for the treatment of uncomplicated or drug-sensitive TB (DS-TB) (World Health Organization [WHO], 2017). The intensive phase is aimed at killing the tubercle bacilli as fast as possible to reduce the bacterial load to stop community transmission whereas the continuous-phase aims for complete sterilization of the slow-growing bacilli (World Health Organization [WHO], 2010). Even though TB is manageable with anti-TB drugs, the emergence of drug-resistant (DR) strains of the *Mycobacterium tuberculosis* complex (MTBC), the causative agent of TB, threatens to make the disease untreatable.

Tuberculosis drug resistance include INH-mono-resistant (INH^r^) for resistance to only INH, RIF-mono-resistant (RIF^r^) for resistance to only RIF (when susceptibility to INH is known), RIF-resistance (RR) for resistance to RIF diagnosed using GeneXpert (when INH susceptibility is not known) multidrug resistance (MDR) for resistance to both INH and RIF. Recently, the definition for extremely drug resistance (XDR), which was MDR with additional resistance to at least one second line aminoglycoside (AMG) and fluoroquinolone (FQ) was changed after the WHO expert consultation (WHO, 2020b) to ‘*TB caused by MTBC strain that fulfils the definition of MDR/RR-TB with additional resistance to any FQ and at least one of bedaquiline (BDQ) and linezolid (LZD)*.’ Similarly, the definition of pre-XDR which was MDR with additional resistance to either any second line FQ or an injectable AMG was also changed to “*TB caused by MTBC strain that fulfils the definition of MDR/RR-TB is also resistant to any FQ*” after the expert consultation meeting (WHO, 2020b). Therefore, polyresistance (PR) TB can then be defined as INH^r^ with additional resistance to at least one second line drug. Treatment of DR-TB requires prolonged administration of relatively more toxic, less efficacious medication (WHO, 2012, 2021) extending the time for sputum conversion. This fuels the spread of resistant strains within the community. It was estimated that 71% of bacteriologically confirmed TB cases (5.8 million) in 2020 were tested for RIF resistance, among which 132,222 (2.3%) were MDR/RIF-TB and 25,681 (0.4%) resistant to at least one of AMGs and FQs (WHO, 2021). The threat of MDR-TB is further exacerbated by the emergence XDR-TB which does not respond to the commonly available TB drugs and requires the use of new formulations and specialized regimen including some of the newest TB drugs such as bedaquiline (BDQ) and linezolid (LZD) depending on the specific resistant profiles.

Previous research studies conducted in Ghana identified several MDR-TB and pre-XDR-TB cases (Homolka et al., 2010; Asante-poku et al., 2015; Otchere et al., 2016; Osei-Wusu et al., 2018a). To the best of our knowledge, these previous studies were not linked to program activities to benefit respective patients. This program-linked activity was initiated to screen difficult-to-treat (DTT) TB patients (sputum-smear non-convertors, failed, relapsed, and known DR-TB patients as well as TB patients with history of TB treatment) through a collaboration between the National Tuberculosis Control Program (NTP) and the Noguchi Memorial Institute for Medical Research (NMIMR), University of Ghana.

## Methodology

### Ethical statement

The protocols and procedures for this study were reviewed and approved by the institutional review board of the NMIMR, with federal-wide assurance number FWA00001824.

### Case definition, inclusion criteria, case recruitment, and sample collection

#### Case definition

Difficult-to-treat tuberculosis patents in this study included TB patients under DOTS management for at least 2 months but still have sputum-smear positive results, relapse TB patients, TB patients with failed treatment, TB patients with history of TB treatment, and known drug resistant TB patients.

#### Inclusion criterion

All DTT-TB cases in eight regions of Ghana including treatment failures, non-converting follow-up patients, relapsed TB patients, retreatment cases, and known DR-TB patients.

#### Exclusion criterion

All other TB cases that did not meet the inclusion criteria.

#### Case recruitment

Informed written consent was sought from all participants unless the participant was illiterate; in which case witnessed oral consent was used. For Children below the age of 18 years, consent from their legal guardians and assent from the children themselves when possible were sought before enrollment. Data collected included age, sex, TB treatment history and treatment outcome from their respective clinical files by following laid-down NTP of Ghana regulations.

#### Specimen collection and transport

Sputum or gastric lavage samples were collected at respective health facilities and transported following the routine Ghana Health Service sputum sample transport algorithm. The samples were triple packaged, sealed, and sent under cold chain to the NMIMR for further analysis.

### Processing of sputum samples

Sputum samples were processed within 24 h upon receipt to maximize isolation of viable MTBC. The sputum samples were transferred into 50 mL falcon tubes and decontaminated using the NALC-NaOH method (Kubica et al., 1963). Briefly, 2X volume of the NALC-NaOH was added to the sputum and vortexed intermittently for 25 mins followed by the addition of 1 normal phosphate buffered saline (PBS) to the 50 mL mark. The resulting suspension was centrifuged at 3,800 rpms for 30 mins followed by decantation of the supernatant. The pellet was resuspended in 2 mL of 1 N PBS and split into two aliquots of 1 mL each for genotypic drug susceptibility testing and archiving for future use.

### Genotypic drug susceptibility testing by line probe assay

The decontaminated sputum aliquot for genotypic DST was heat-inactivated at 95°C for 60 min followed by a quick DNA extraction using the genolyse method (Hain Lifescience) according to the manufacturer’s instruction. The extracted DNA was screened for susceptibility to INH and RIF using the Genotype MTBDR*plus* (Hain lifescience) according to the manufacturer’s protocol. Those resistant to RIF and INH as well as RIF mono-resistant samples were screened further by Genotype MTBDR*sl.v2* (Hain Lifescience) for susceptibility or otherwise to the second line FQs and AMGs following the manufacturer’s protocol.

## Results

### Patient’s demography

**FIGURE 1:**
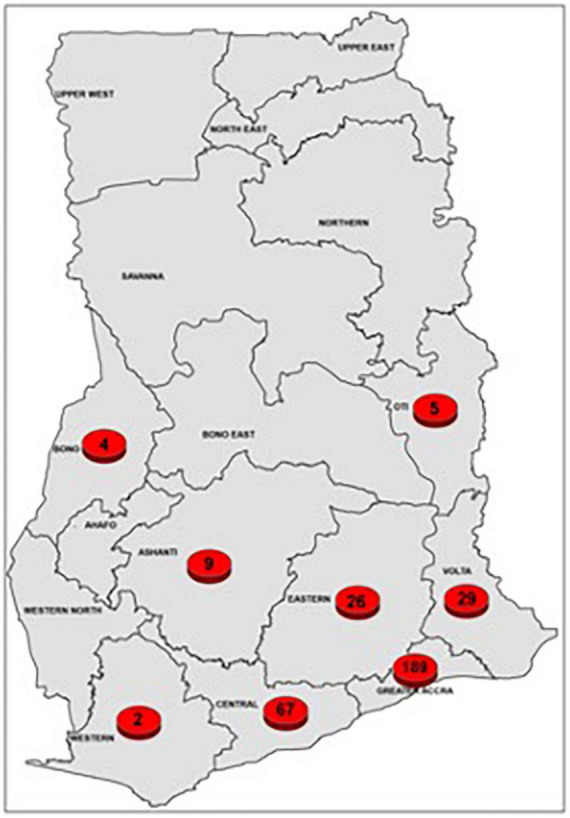
Regional distribution of recruited patients for the study.

**FIGURE 2:**
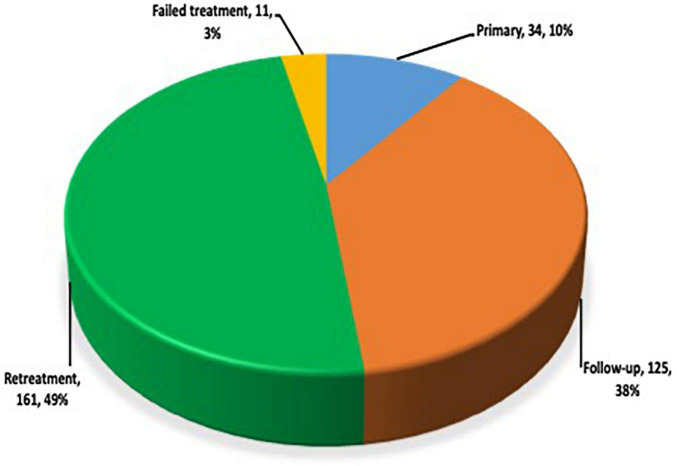
Distribution of study participants by patient category.

Samples from 331 DTT-TB patients comprising 112 (33.8%) females and 219 (66.2%) males were received between August 2017 and October 2019. The age of the participants ranged from 1 to 82 years with a median age of 42 years. The participants were from 8 out of the 16 regions of Ghana, including Ashanti (9), Bono (4), Central (67), Eastern (26), Greater Accra (189), Volta (29), Oti (5), and Western (2) as shown below (Figure 1). Most of the study participants (*n* = 161, 48.6%) were retreatment cases, 2- and 5-months follow-up cases (125, 37.8%), newly diagnosed DR-TB contacts or were RIF-positive by GeneXpert 34 (10.3%), and lastly those with failed treatment 11 (3.3%) (Figure 2).

### Resistance to first-line anti-TB drugs and observed pattern of resistance

Out of the 331 sputum samples processed, the MTBDR*plus* assay detected MTBC DNA in 298 (90%). There were 123 (41.3%) drug-susceptible (DS) samples and 175 (58.7%) samples resistant to at least one drug (ANY^r^). 50 (16.8%) were resistant to only INH (INH^r^), 50 (16.8%) resistant to only RIF (RIF^r^), and 75 (25.2%) resistant to both INH and RIF (MDR). Stratified by patient category (Table 1), we found many new TB cases under follow-up (90%) were sensitive to both INH and RIF (OR = 44.2, 95%CI = 20.3–105.2, *p* < 0.0001), whereas most of the retreatment and failed treatment TB cases, respectively, were MDRs (35.1%) and INH^r^ (50%). Retreatment cases (54/154) were more likely to be MDR (OR = 3.2, 95%CI = 1.7–5.9, *p* < 0.0001). Strikingly, two of the 50 RIF^r^ samples came from new TB cases (Supplementary Table 1).

**TABLE 1:** Distribution of first line anti-TB drug resistance among 298 difficult-to-treat (DTT) tuberculosis (TB) cases.

Patient category (Number of patients)	Sensitive 123 (41.3%)	INH^r^ 50 (16.8%)	RIF^r^ 50 (16.8%)	MDR 75 (25.2%)	ANY^r^ 175 (58.7%)
Known DR-TB (34)	-	-	17 (50.0%)	17 (50.0%)	34 (100%)
Follow-ups (100)	90 (90.0%)	6 (6.0%)	2 (2.0%)	2 (2.0%)	10 (10%)
Retreatment (154)	30 (19.5%)	39 (25.3%)	31 (20.1%)	54 (35.1%)	124 (80.5%)
Failed treatment (10)	3 (30.0%)	5 (50.0%)	-	2 (20.0%)	7 (70%)

INH^r^, isoniazid monoresistance; RIF^r^, rifampicin monoresistance.

From the MTBDR*plus* assay band patterns (Table 2A), we observed that among the 50 INH^r^ samples, 30 (60%) carried *katG* mutations (MT), 4 (8%) *inhApro* MT, and 16 (32%) both *katG* and *inhApro* MT. Most *katG* mutant INH^r^ samples had both absence of wild-type (WT) bands and presence of mutation bands (93.3%), whereas the presence of mutation bands was the main occurrence seen among *inhApro* mutant INH^r^ samples (75%). The 50 RIF^r^ samples had *rpoB* MT including 15 (30%) missing WT bands, 27 (54%) presence of MT bands and 8 (16%) both absence of WT bands and presence of mutation bands. The 75 MDR samples included 41 (54.7%) due to *katG* and *rpoB* MT, 11 (14.7%) because of *inhApro* and *rpoB* MT and 23 (30.6%) due to both *katG* and *inhApro* MT. Dual absence of WT bands with accompanying presence of mutation bands was the major occurrence (78.3%) among the triad *katG*, *inhApro*, and *rpoB* mutant MDR samples.

**TABLE 2A:** Observed expanded MTBDR*plus* band patterns.

Drug resistant	Locus	WT band absent	MT band present	WT band absent and MT band present
INH^r^ (50)	*katG* (30;60%)	0 (0%)	2 (6.7%)	28 (93.3%)
	*inhApro* (4;8%)	0 (0%)	3 (75.0%)	1 (25.0%)
	*katG* + *inhApro* (16;32%)	0 (0%)	1 (6.3%)	15 (93.7%)
RIF^r^ (50)	*rpoB* (50;100%)	15 (30.0%)	27 (54.0%)	8 (16.0%)
MDR (75)	*katG* + *rpoB* (41;54.7%)	5 (12.2%)	17 (41.5%)	19 (46.3%)
	*inhApro* + *rpoB* (11;14.7%)	2 (18.2%)	5 (45.5%)	4 (36.4%)
	*katG* + *inhApro* + *rpoB* (23;30.7%)	0 (0%)	5 (21.7%)	18 (78.3%)

WT, wild-type; MT, mutant. WT band absent represents disappearance of an expected WT band as marker for drug resistance, MT band present represents observation of a mutation band as a marker for drug resistance whereas WT band absent and MT band present represent a unique observation of a missing WT band in addition to an appearance of a mutation for the same sample as determinant of drug resistant.

Seventy-one (60.0%) of INH-resistant samples (including INH^r^ and MDR) had *katG* MT, whereas 15 (12%) had *inhApro*-MT (Table 2B). The remaining 39 (31.2%) had both *katG* and *inhA* MT. Comparing the *katG* locus and *rpoB*, respectively, as the major contributors of INH and RIF resistance, we discovered that *katG* mutants were more likely (*p* < 0.0001) to have missing WT band with corresponding mutation band.

**TABLE 2B:** Observed condensed MTBDR*plus* band patterns.

Drug resistant	Locus	WT band absent	MT band present	WT band absent and MT band present
INH (125)	*katG* (71;56.8%)	5 (7.0%)	19 (26.8%)	47 (66.2%)
	*inhApro* (15;12.0%)	2 (13.3%)	8 (53.3%)	5 (33.3%)
	*katG* + *inhApro* (39;31.2%)	0 (0%)	6 (6.3%)	33 (84.6%)
**RIF (152)**	*rpoB* (125)	22 (17.6%)	54 (43.2%)	49 (39.2%)

### Resistance to second-line anti-TB drugs and observed resistance pattern

We further screened the 175 first-line RIF resistant samples (including the 75 MDRs, 50 INH^r^ and 50 RIF^r^) with the MTBDR*sl* for resistance to FQs and injectable AMGs (Table 3). We found 151 (86.3%) of the samples were susceptible to both FQS and AMGs whereas 24 (13.7%) were resistant to at least one of the second-line drugs. These included 7.4% (2 RIF^r^, 1 INH^r^, and 10 MDR samples) resistant to only FQs and 2.3% (2 RIF^r^, 1 INH^r^, and 1 MDR samples) resistant to AMG drugs kanamycin (KAN), amikacin (AMK), capreomycin (CAP), and viomycin (VIO). Additionally, there were 4.0% (5 RIF^r^ and 2 MDR samples) resistant to both FQs and AMGs comprising 4 RIF^r^ (including the two new TB samples) resistant to FQs and AMG drugs KAN/AMK/CAP as well as 1 RIF^r^ and 2 MDR samples with additional FQ resistance and low-level KAN resistance. Using the current WHO definitions (WHO, 2020b), we identified 19 pre-XDR-TB samples (MDR/RR-TB samples with additional resistance to FQs) and 5 polyresistant samples (2 INH^r^ and 3 MDR/RR-TB samples with additional resistance to AMGs).

**TABLE 3:** Second-line tuberculosis (TB) drug susceptibility profile of 175 first-line TB drug resistant samples.

First line resistance	Second line drug profile										
	151 (86.3%) Susceptible	13 (7.4%) FQs	4 (2.3%) AMGs				7 (4.0%) AMGs + FQs				ANY^r^ 24 (13.7%)
			KAN/AMK/CAP	KAN/CAP/VIO	KAN/AMK/CAP/VIO	Low-level KAN	KAN/AMK/CAP	KAN/CAP/VIO	KAN/AMK/CAP/VIO	Low-level KAN	
RIF^r^ (50)	41 (82.0%)	2 (4.0%)	-	-	2 (4.0%)	-	4 (8.0%)	-	-	1 (2.0%)	9 (18.0%)
INH^r^ (50)	48 (96.0%)	1 (2.0%)	-	-	1 (2.0%)	-	-	-	-	-	2 (4.0%)
MDR (75)	62 (82.7%)	10 (13.3%)	-	-	1 (1.3%)	-	0	-	-	2 (2.7%)	13 (17.3%)

Among samples resistant to at least one second-line anti-TB drug (Table 4), the mutants were either *gyrA, rrs* or *eis*, and/or combinations thereof. There were three samples with absence of WT *gyrA* bands and 10 with present *gyrA* mutation bands among the FQs resistant samples. There were four samples with only *rrs* Mut2 band corresponding to KAN/AMK/CAP/VIO resistance. Additionally, there were seven samples with *gyrA* Mut3B band, three of which had *eis* Mut1 band conferring low-level KAN resistance whereas the remaining four had the *eis* Mut1 and rrs Mut1 bands conferring resistance to KAN, AMK, and CAP. All the 20 samples resistant to FQs were *gyrA* mutants whereas the 11 samples resistant to AMGs were *rrs* (4) and *eis* (3) as well as *rrs*, and *eis* co-mutants (4). Except for seven pre-XDR samples (2 MDR and 5 RR), no sample had *eis* MT (Supplementary Table 1).

**TABLE 4:** Observed MTBDR*sl* assay band patterns.

Group	Locus	WT band absent	MT band present	Resistance
FQs (13)	*gyrA*	3 (1 *gyrA* WT1 and 2 *gyrA* WT3)	10 (3 *gryA* Mut1, 1 *gryA* Mut3A and 6 *gyrA* Mut3B)	FQs
AMGs (4)	*rrs*	-	4 (*rrs* Mut2)	KAN/AMK/CAP/VIO
FQs + AMGs (7)	*gyrA* + *eis*	-	3 (*gryA* Mut3B and *eis* Mut1)	FQs + low-level KAN
	*gyrA* + *eis* + *rrs*	-	4 (*gryA* Mut3B, *eis* Mut1 and *rrs* Mut1)	FQS + KAN/AMK/CAP

## Discussion

Resistance to anti-tuberculosis drugs is a major threat to achieving the End TB goals (WHO, 2012; Getahun et al., 2013; El Hamdouni et al., 2019). Early diagnosis of TB and ascertaining the drug susceptibility profile of the infecting pathogen are required to initiate an appropriate treatment regimen. However, most TB management protocols around the globe are based on passive case detection leading to late diagnosis with its associated inimical public health implications. We passively screened TB patients in Ghana belonging to at least one of the groups including retreatment, failed treatment (sputum non-converting), defaulted treatment, and known DR-TB.

We found that 58% of cases were resistant to at least one first-line drug (ANY^r^) including 16% INH^r^, 16.8% RIF^r^, and 25.2% MDR. These proportions are significantly higher than what was detected by previous independent studies in Ghana (Otchere et al., 2016; Addo et al., 2017). However, our findings compares with findings from a recent study in Ghana (Sylverken et al., 2021) and others parts of the world (He et al., 2008; Oxlade et al., 2010; World Health Organisation and Regional Office, 2014; Luo et al., 2017; Song et al., 2020; Mbuh et al., 2021) which reported higher MDR rates among retreatment cases compared to new TB cases. New TB cases among the DTT-TB samples were more likely to be susceptible to INH and RIF (*p* < 0.001) compared to retreatment cases suggesting that higher bacteria load may be the cause of sputum non-conversion after the intensive phase of DOTS but not drug resistance. Such cases normally require extension of the intensive phase of the treatment regimen by at least on month for better clearance of the bacteria (World Health Organization [WHO], 2010).

Our study confirmed *katG* MT as the main cause of INH-resistance in Ghana, and these samples were more likely to have absence of WT bands with corresponding presence of MT bands compared to *rpoB* mutant RIF-resistant samples which mostly had presence of MT bands without missing WT bands. This observation suggests that most of the affected TB patients had INH-resistance already fixed in the infecting bacteria while RR had nascently emerged among a sub-population of the bacteria. This finding support the general idea that INH-resistance is the precursor for emergence of MDR-TB (Stagg et al., 2017; O’Donnell, 2018; Srinivasan et al., 2020). Unfortunately, the most recommended rapid TB diagnostic tool (GeneXpert) only detects RR as proxy for MDR-TB. There is therefore the need to consider incorporating detection of INH-resistance into the GeneXpert and/or any other new diagnostic tool to aid in early detection of INH-resistance to curb emergence of MDR-TB.

The MTBDR*sl* assay targets MT within the “*quinolone resistance-determining regions*” spanning the *gyrA* and *gyrB* loci thus increasing its sensitivity (Sun et al., 2008). We found from our study that all FQ-resistant samples had either *gyrA* WT band missing or *gyrA* mutation band present or both with no involvement of the *gyrB* locus. This observation agrees with the findings of two independent studies (Von Groll et al., 2009; Kabir et al., 2020) which found over 98% of FQ-resistance was caused by *gyrA* MT alone. The *gyrA* and *gyrB* are the two sub-units of the gyrase enzyme which is the target of FQ drugs (Xu et al., 1996). Therefore, the observation of only *gyrA* MT among FQ-resistant samples is mind bugling. Could it be that the *gyrA* sub-unit of the bacterial DNA gyrase is potentially the main target of FQ drug or do *gyrA* MT offer competitive advantage to the bacteria?

There were 2 out of the identified 19 pre-XDR samples which would have been classified as XDR-TB samples by the old WHO definition (MDR with further resistance to FQs and AMGs). These samples had genetic alterations at the *gyrA* and *eis* loci (for low-level KAN resistance) compared to the first XDR-TB case identified in Ghana (by the old WHO definition) which had MT at the *rrs* loci (for resistance to KAN/AMK/CAP/VIO) and *gyrA* (Osei-Wusu et al., 2018b). These two scenarios clearly have different strains of MTBC which acquired resistance through different evolutionary paths and would require different combination of drugs for treatment (Kambli et al., 2016). Furthermore, the new WHO definitions of XDR and pre-XDR TB (WHO, 2020b) highlight the importance of FQs but not AMGs in the management of MDR/RR TB and the need to prevent emergence and potential spread of FQ resistance which is the precursor for pre-XDR and XDR TB among MDR/RR TB patients. Nevertheless, all the identified AMG-polyresistant samples were associated with the *rrs* locus without the involvement of the *eis* locus, suggesting high level resistance to AMGs (Kambli et al., 2016) which cannot be ignored. Nevertheless, the identification of two pre-XDR TB cases at primary diagnosis (indication of potential ongoing transmission of pre-XDR TB) from two different regions of the country is very alarming.

## Conclusion

We report the detection of several pre-XDR TB cases in Ghana through passive screening of DTT TB patients. Our study is limited by the fact that we did not have any rapid diagnostic tool for detection of BDQ and/or LZD resistance to determine whether some of the identified 19 pre-XDR samples are XDR-TB samples by the new WHO definition. Nevertheless, the high proportion of second line resistance among retreatment cases call for intensified surveillance and active monitoring of TB patients, especially those at risk of drug resistance. Additionally, the likelihood of an ongoing transmission of pre-XDR TB in Ghana calls for active surveillance for DR-TB. There is also the need for inclusion of BDQ and LZD in existing and new rapid diagnostics to enhance early detection to support an appropriate treatment regimen to help curb the spread of DR-TB. Finally, there is the need for intensified public education on the need to comply with TB treatment regimen for effective treatment toward reduction of DR-TB.

## Data availability statement

The original contributions presented in this study are included in the article/Supplementary material, further inquiries can be directed to the corresponding author.

## Ethics statement

The protocols and procedures for this study were reviewed and approved by the institutional review board of the NMIMR, with federal-wide assurance number FWA00001824. Written informed consent to participate in this study was provided by the participants’ legal guardian/next of kin.

## Author contributions

IO conceptualized the idea, developed methods, provided resources, carried out the research, wrote first draft, and reviewed the manuscript. PM, PA, SO-W, SA, AM, and TA carried out the research and reviewed the manuscript. SY, ED, GT-O, CL, YP, and FB provided resources and reviewed the manuscript. AA-P provided resources, carried out the research, and reviewed the manuscript. SG provided resources, supervised the work, and reviewed the manuscript. DY-M conceptualized the idea, developed methods, provided resources, supervised the work, and reviewed the manuscript. All authors contributed to the article and approved the submitted version.
